# Motor–Cognitive Associations in Older Adults: A Cross-Sectional Study Toward Self-Assessment Tools

**DOI:** 10.3390/bs16020291

**Published:** 2026-02-18

**Authors:** Hwang Jin, Tianpei Li, Chulwook Park

**Affiliations:** 1 Department of Physical Education, Jeonbuk National University, Jeonju 54896, Republic of Korea; jeanh@jbnu.ac.kr (H.J.); tianpeili9611@163.com (T.L.); 2 Department of Physical Education, Seoul National University, Seoul 08826, Republic of Korea; 3 Complexity Science and Evolution, Okinawa Institute of Science and Technology, Okinawa m1919-1, Japan; 4 Systemic Risk and Resilience, International Institute for Applied Systems Analysis, 2361 Laxenburg, Austria

**Keywords:** cognitive aging, motor coordination, Stroop test, self-diagnostic tools, executive functions

## Abstract

Background: This study explored the interrelation between motor coordination abilities and cognitive functions in older adults, aiming to establish a preliminary diagnostic tool that may facilitate early detection of motor–cognitive decline. Methods: Utilizing a mixed-methods approach, we investigated the efficacy of the Stroop word test in conjunction with various motor coordination measurements to identify markers of cognitive aging in older adults. Results: The analysis revealed significant correlations between asymmetric spatial coordination (AC) and Stroop error effects (SEEs), indicating that better coordination correlates with reduced cognitive errors. Multiple-regression analysis showed that AC, simple reaction time (SRT), and anticipation time (AT) significantly predicted SEE (R^2^ = 0.635), with AC emerging as the strongest predictor (β = −0.475). These results underscore the significance of asymmetric spatial motor coordination as a predictive factor for executive cognitive abilities affected by aging. We propose a potential tool for individuals to monitor their motor–cognitive health. Conclusions: The findings of this study contribute to the growing body of evidence linking physical coordination to cognitive function, emphasizing the importance of integrated diagnostic approaches in the management of aging-related cognitive impairments.

## 1. Background

As awareness of health and welfare has recently increased, so has the interest in quality of life. The definition of health has expanded from merely being “free from illness” to a state of “physical, mental, and social well-being,” thereby occupying a crucial position in modern society (Office of Disease Prevention and Health Promotion, 2010). Research has reported that regular physical activity positively affects the realization of a welfare society and is closely linked to the improvement of individuals’ quality of life, emphasizing the importance of physical activity in order to maintain a healthy life for as long as possible (Fontaine et al., 2003).

This perspective, along with advancements in neuroscience, necessitates the application of neuroscience methodologies to exercise behavior. In particular, research on the cognitive and neurological changes involved in movement as they relate to aging is being conducted in various fields (Hillman et al., 2006). The interaction between the environment, tasks, and organisms involved in aging has highlighted the substantial role of the nervous system, without which smooth functioning of perception, cognition, and the musculoskeletal system is impossible (Carson & Kelso, 2004). Consequently, the need for neuromotor research related to aging has increased (Voelcker-Rehage et al., 2010).

Accordingly, this study aimed to comprehensively characterize motor control abilities among older adults based on the movement and cognitive control characteristics found in existing experiments and research. By determining the impact of integrated motor control abilities in older adults on the use of cognitive functions, we sought to examine in detail the neuromotor control characteristics induced by aging. Additionally, by simplifying the complex and varied measurement equipment and technologies into a single diagnostic tool that individuals can use, we aimed to make it easier to ascertain the degree of aging.

As the global population ages, diseases related to older adults have become a critical social issue. The transformation into an aging society has resulted in a growing older adult population (Harper, 2014; UN DESA, 2019), with many countries, including South Korea, projected to become super-aged societies in the coming decades. This phenomenon reflects middle-aged and older adults’ increasing need to engage in meaningful life activities during their senior years as the extension of their lifespan results in a higher proportion of older adults in the population (Hunter et al., 2016). Consequently, it is vital to develop specialized knowledge services, including functional-problem diagnosis and prevention technologies, to enrich the lives of older adults. Health issues among older adults can lead to an increased societal burden through medical costs, which suggests that suitable diagnostic and preventative technologies could have considerable economic benefits in the long term (Booth & Lees, 2006).

Human brain activity is closely connected to various behaviors (Kandel et al., 2000), and exercise significantly improves cognitive functions (Kim et al., 2007; Burpee & Stroll, 1936). This positive relationship extends to the older adult population; despite age-related declines in information processing speed and short-term memory (Castelli et al., 2007; Bäckman et al., 2010; Cabeza, 2001; Lövdén et al., 2010; D. C. Park & Reuter-Lorenz, 2009), significant differences in cognitive functions have been documented in groups participating in exercise (S. Colcombe & Kramer, 2003). Continuous exercise enhances cardiovascular function, physical balance, and muscle strength in older adults (Fatouros et al., 2002), while also stimulating the nervous system through increased blood flow to the brain, positively affecting brain function activation (Spirduso, 1980). This suggests a beneficial relationship between older adults’ exercise capabilities and cognitive function (S. J. Colcombe et al., 2004). Neurophysiological evidence supports the importance of physical activity in maintaining cognitive abilities in older adults, including angiogenesis, construction of new neural fibers, and adaptability of neurotransmitter systems (Black et al., 1990; van Praag et al., 2005). These mechanisms make cardiovascular training beneficial for cognitive function and show positive effects on brain cortical areas related to attention control (S. Colcombe & Kramer, 2003; Lustig et al., 2009).

The importance of physical activity in retaining and developing cognitive abilities in healthy older adult individuals (e.g., S. Colcombe & Kramer, 2003) has expanded from the classical perspective of the neurophysiological plasticity of cardiovascular exercise (Hillman et al., 2008) to include motor fitness aspects such as movement speed, balance maintenance, and coordination (Stojan et al., 2023; Voelcker-Rehage et al., 2010). In particular, various forms of movement coordination linked to daily activities are closely associated with the neurological activities of older adults, presenting evidence that coordination indirectly and directly affects not only their activities and lifestyles in old age but also their self-esteem and cognitive functions (Rosenbaum, 2005). This suggests that various forms of coordinated movement can serve as a foundation for determining older adults’ behavioral health habits, potentially acting as a basis for reducing the healthcare costs associated with aging (Rinn et al., 2023).

This cross-sectional study, thus, aimed to (1) examine associations between motor coordination abilities (temporal and spatial measures) and cognitive function (Stroop test performance) in community-dwelling older adults, and (2) develop a preliminary self-assessment model that could facilitate early identification of motor–cognitive decline. We hypothesized that different coordination tasks, such as (spatial–)temporal and (a)symmetric, would show different associations with executive function measures.

## 2. Methods

### 2.1. Motor (Coordination) Function in Older Adults

**Figure 1:**
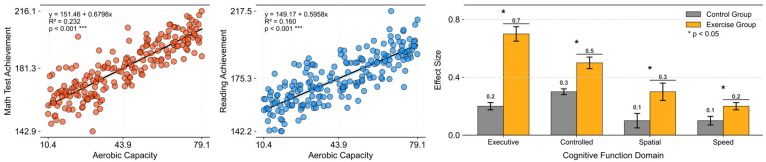
Recreated effects of physical activity in cognitive function. **Left** = physical activity and academic performance in school-age children (data from the California Department of Education); **right** = meta-analytic findings of exercise-training effects on cognition in older adults (data from S. Colcombe & Kramer, 2003).

Owing to the newly discovered effects of exercise on cognitive function, research on the relationship between exercise and cognition has begun to attract attention from various perspectives. Significant positive effects have been observed not only in academic achievement but also in aging, which has recently become a major social issue (Trudeau & Shephard, 2008) (Figure 1).

Although most studies have acknowledged the relationship between exercise and cognitive function, their findings have typically been limited to health-related physical fitness components. This approach has been criticized for overlooking the diverse effects of exercise by focusing solely on cardiorespiratory fitness aspects (Voelcker-Rehage et al., 2010). Notably, human motor behavior involves not only improvements in cardiorespiratory function and muscle strength, but also functional enhancements that cannot be fully explained by structural, anatomical, and physiological changes in the brain (Hillman et al., 2008).

### 2.2. Spatial Coordination Variables

Reports have indicated that aerobic and coordination exercises produce different enhancements in the cognitive functions of older adults. Coordination exercises are closely related to executive functions associated with attention, memory, spatial–temporal organization and maintenance, self-regulation, continuous action, flexibility, response inhibition, and planning (Kaplan et al., 2000). Therefore, the relationship between exercise and cognition may be examined from various angles to demonstrate the relationships among the acquisition of motor coordination, motor skills, and cognitive functions (Shi et al., 2020). However, the tools used to measure exercise coordination characteristics have methodological limitations. Based on classifications by Henry (1961), Fleishman (1972), Keele et al. (1987), and others, most prior studies have been limited to time-dependent motor responses, which limits the discussion on coordination details. Time-dependent measures commonly used in previous research (e.g., movement rate = speed of hand and foot movements) are limited in their ability to assess the coordination of limbs moving efficiently together. Accordingly, to verify the aging effects related to various motor functional abilities, this study measured not only the time-dependent eye and hand coordination used in previous studies, but also spatially dependent limb (a)symmetry coordination (Haken et al., 1985; C. Park, 2022) (Table 1). (See Appendix A.1 for details of the model.)

### 2.3. Reaction Time Variable

Information received from the external environment enables behavioral regulation through appropriate neural actions. Neural activity is a primary factor regulating human movement, and studies related to the action of nerves have traditionally used reaction time (simple reaction time) as a measure (Fiani et al., 2021). Reaction times can vary substantially depending on the nature of the task and individual differences (Hick, 1952). Anticipation, which involves controlling the initiation of movement in response to external stimuli, plays a critical role in timing. If performed correctly, it can considerably improve efficiency in terms of timing, accuracy, and force applied. Further, having more time to process information through anticipation can enhance efficiency (Schmidt et al., 2018). From everyday actions (e.g., walking and running) to complex sports skills (e.g., swinging a golf club and performing gymnastics flips) all human movements require harmonious connections between physical elements (e.g., joints and muscles). Creating forms for various actions necessary in daily life and understanding the principles of movement coordination (Bernstein, 1967) and the process of change to achieve performance goals more effectively are the first steps in uncovering the information needed to understand aging. Such measurements are expected to not only validate the reliability of time-dependent measurement tools (simple reactions) used in previous studies aiming to explore the mechanisms of aging, but also provide a clearer understanding of the aging state of motor abilities through comparisons with spatially dependent limb coordination (inter-limb coordination) capabilities.

### 2.4. Cognitive (Executive) Function Measures

**Figure 2:**
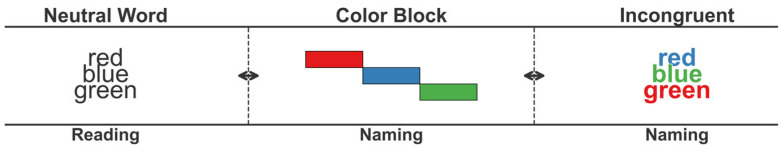
Prototype of the task. Note: left = prototype of the Stroop task: Stroop 1 = reading the word, Stroop 2 = reading the color, Stroop 3 = reading the color and not the word (referenced from Keele, 1972; Hecht & Reiner, 2010).

The Stroop task, which involves parallel processing of a color and the word representing the color and performing an interference, is an excellent tool for providing insights into interference and facilitation within the sensory-perception–action loop (Keele, 1972). The Stroop task is one of the most frequently used tools for measuring executive functioning in older adults (Hecht & Reiner, 2010) as it examines mental flexibility and the ability to suppress preferred responses when performing new actions (Kaplan et al., 2000), which is related to the demands of frontal-lobe function in extracting appropriate information, recognizing it, and controlling interferences (Stuss & Benson, 1986) (Figure 2).

Executive function refers to the ability to self-regulate, act continuously, exercise flexibility, respond to inhibition and planning, and appropriately organize one’s behavior (Friedman & Robbins, 2022). It differs from general cognitive abilities (e.g., attention and memory), primarily involving conscious neural regulation performed in the prefrontal cortex, which includes the integration, organization, maintenance, and flexibility of behavior (American Psychological Association, 2000; Kaplan et al., 2000). Executive function significantly correlates with age, and studies comparing executive function tasks across various age groups have reported clear differences in task scores with age. However, most research findings have limitations when applied to domestic situations due to certain variables, including sample selection criteria, experimental methods, and individual characteristics (education, attention, and memory abilities). Considering the limitations concerning the Stroop test in previous studies, this study sought to validate the correlations between variables in an experimental environment (Gajewski et al., 2020). To investigate the effects of age, gender, and education level on performance in the Stroop test as standard measurement data, we compared the different coordination effects related to Stroop test performance scores among older adult volunteers. As indicated in Appendix A.2, the Stroop test comprises three subtasks. We instructed participants to read the tasks included in each performance as quickly as possible without feeling pressured by time constraints. We measured the performance time for Tasks 1, 2, and 3, the interference (Stroop 3 − [(Stroop 1 + Stroop 2)/2]), and the frequency of errors. This formula isolates the interference effect by comparing incongruent trial performance to the average of congruent conditions, following standard procedures (MacLeod, 1991).

### 2.5. Participants and Recruitment Process

A total of 30 elderly participants (20 females, 10 males) took part in this cross-sectional experiment. The average age was 80 years (SD = 5, range 71–89). On average, participants had 11.3 years of education (SD = 2.77, range 9–16). They engaged in leisure and physical exercise activities for an average of 6.5 h per week (SD = 2.02, range 5–10). The average body mass index (BMI) was 23.85 kg/m^2^ (SD = 1.06). The average systolic blood pressure was 140.30 mm Hg (SD = 3.22) and diastolic blood pressure was 82.38 mm Hg (SD = 2.01).

All participants volunteered for the study and were screened for medical issues and health restrictions. Recruitment was done through the member registry of a college program for the elderly. Medical exams included assessments of general motor (cardiovascular, osteoarthritis, and arthrosis) and neurological functions (brain disorders, Parkinson’s disease, and stroke), which found no significant symptoms. Three participants withdrew (two males, one female). Additionally, three participants (two males, one female) were identified as outliers due to z-scores exceeding ±2.5 standard deviations from the mean (Ratcliff, 1993), with extreme values for SE or SEE (see Appendix A.2.2 for details), and were excluded from the analysis. Thus, data from 24 participants were analyzed.

Testing was conducted at a community center equipped with standardized laboratory devices and a computerized measurement system. Each participant completed all motor coordination tasks (SRT, AT, SC, AC) followed by the Stroop test in a single session, with brief rest periods provided between tasks as needed. All statistical analyses were performed using IBM SPSS Statistics (Version 29.0). Descriptive statistics, Pearson correlations, and multiple-regression analyses were conducted, with statistical significance set at *p* < 0.05.

The participants provided written informed consent to participate in this study (Clinical trial number: not applicable). The study was approved by the local ethics committee (SNUIRB No. 1509/002-002) and adhered to the ethical standards of the 1964 Declaration of Helsinki (CITI Program Record ID 20481572).

## 3. Results

### 3.1. Motor (Coordination) Function

As presented in Table 2, we analyzed the descriptive statistics for each variable, including simple reaction time (SRT), anticipation time (AT), Symmetrical Spatial Coordination Ability (SC), and Asymmetrical Spatial Coordination Ability (AC). The total number of participants in the experiment was 24 (after exclusions), of whom three were deemed outliers based on the criterion of quartile deviation from the mean value proposed by Ratcliff (1993) and were excluded from the analysis. The sex composition included both males and females, with ages ranging from 71 to 82 yrs. (see Appendix A.2.1 for more details.)

Examination of the mean (M) values and standard deviation (SD) for each variable showed that the average simple reaction time (SRT) was 7.93 s, with a standard deviation of 0.84 s. Anticipation Time (AT) was 2.51 s on average, with a standard deviation of 1.10 s. The index for Symmetrical Spatial Coordination Ability (SC) was 1.34 on average, with a standard deviation of 0.21, whereas that for Asymmetrical Spatial Coordination Ability (AC) was 0.36 on average, with a standard deviation of 0.24. Thus, the time- and space-based coordination abilities of participants varied significantly.

**Figure 3:**
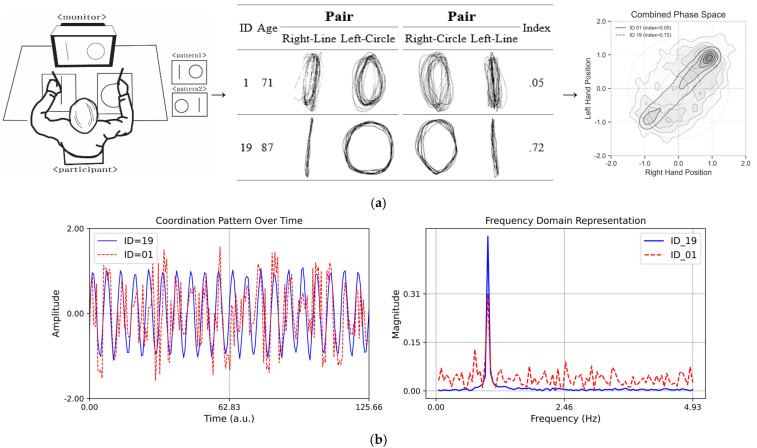
(**a**) Schematic drawing of the asymmetrical bi-manual coordination tasks. (Left) A perfect independent would have a higher index score (perfect score = 1.0), and the worst independent would have a lower index score (worst score = 0.0). (Middle) Asymmetrical bi-manual drawing displacement in millimeters: the X (major) and Y (minor) axis dimensions of lines and circles when subjects performed dual tasks for a 20 s trial for each pair condition. (Right) Combined phase space representation of coordination dynamics showing density distributions for participants with contrasting stability indices. (**b**) Coordination patterns over time. The plot on the left side displays two sinusoidal time series with variations in different coordination complexities. The blue line (ID = 19, index = 0.72) represents stable coordination, while the red dashed line (ID = 01, index = 0.05) exhibits high variability, illustrating complexity. The plot on the right side presents the Fourier transform of the time series, highlighting the differences in frequency content. The red dashed line shows a broader frequency spectrum, indicating greater complexity compared to the more uniform blue line.

In particular, as depicted in Figure 3a, Asymmetrical Spatial Coordination Ability showed high variability in both average reaction times and indices, which suggests the presence of individual differences in cognitive and motor function during aging. The comparison in Figure 3b highlights the difference between the selected prototypical cases (ID = 19, index = 0.72; ID = 01, index = 0.05), which is useful for motor control and coordination pattern recognition. The results obtained through the analysis, excluding outliers, provide important insights for research on the motor and cognitive functions of the older adult population.

### 3.2. Cognitive (Executive) Function

Table 3 presents the descriptive statistics for executive function, cognitive function, the Stroop effect (SE), and the Stroop error effect (SEE) (data on participants’ sex, age, and cognitive function indicators). Three participants were excluded from the analysis based on outlier criteria using the quartile deviation from the mean value proposed by Ratcliff (1993). One participant scored 366.04 s (Z: 4.10) on the SE; the second scored 33.5 f (Z: 2.53) on the SEE, and the third scored 36.5 f (Z: 2.85) on the SEE. Owing to the possibility that they may not have come from the same population, they were excluded from the analysis. The exclusion rate (12.5%) is within an acceptable range for reaction time studies (Ratcliff, 1993), and preliminary inspection of the data indicated that the primary AC-SEE correlation pattern remained consistent in direction when examined with the full dataset, supporting the robustness of the findings.

The analysis results showed an average Stroop effect (SE) of 82.31 s (SD = 35.67) and an average Stroop error effect (SEE) frequency of 9.08 (SD = 6.31). These results provide an important foundation for exploring the relationship between SE and SEE in this older adult sample, and provide important insight for exploring changes in executive function during aging and the relationship between SE and SEE. In particular, the high SD for executive function scores and the frequency of SE and SEE suggested significant variability among the participants, which we interpreted as an important consideration in the study of cognitive function in the older adult population. Appendix A.2.2 provides detailed score information for all participants. The results obtained through this analysis, after excluding outliers, contribute to deepening our understanding of cognitive functions and SEE among older adults.

### 3.3. Correlation Between Motor and Cognitive Functions

The primary finding was a significant negative correlation between asymmetric spatial coordination (AC) and Stroop error effects (SEE) (r = −0.475, *p* = 0.033), indicating that better coordination was associated with fewer cognitive errors. To examine this and other motor–cognitive relationships, we analyzed the interrelationship between cognitive function measures (Stroop test) and various temporal and spatial coordination measures. Our aim was to identify the motor components most strongly associated with cognitive performance.

**Figure 4:**
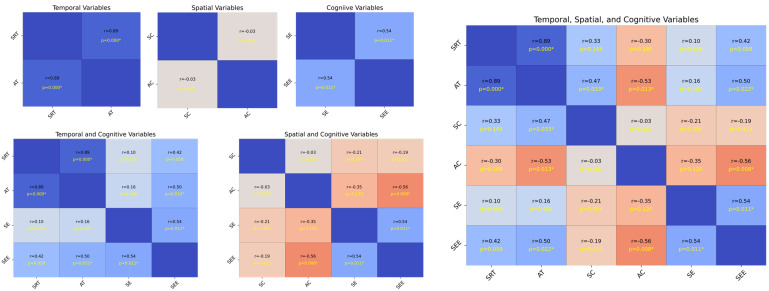
Analysis of standardized variables. The plot on the upper left side consists of SRT, AT, SC, AC, SE, and SEE, and the plot on the bottom left side consists of SRT, AT, SE, SEE, SC, AC, SE, and SEE, while the plot on the right side includes all of them. Each cell shows the linear regression, with Pearson’s correlation coefficients (r) and *p*-values indicating the strength and statistical significance of the relationships, respectively. The scatter plots illustrate the pairwise relationships, and asterisks next to the *p*-values in the upper triangle denote the significance levels, *p* = < 0.05, *p* < 0.01, and *p* < 0.001, respectively. The regression varies according to the significance, assisting in establishing the correlation strength between variable pairs. The presence of significant correlations highlights potential predictive relationships, whereas non-significant correlations (no asterisks) indicate a lack of evidence for a linear predictive relationship between the respective pairs of variables.

Figure 4 illustrates the strength and statistical significance of the correlations between the standardized variables through linear regression analysis, Pearson’s correlation coefficient (r), and *p*-values. The analysis was divided into six subsets, each representing a specific variable category: temporal (SRT and AT), spatial (SC and AC), and cognitive (SE and SEE). The first subset analyzed the temporal variables SRT and AT. The results, along with the linear regression, illustrate the relationships between the variables. The second subset focused on the spatial variables SC and AC. The correlation matrix shows the strength and significance of the linear relationships. The regression analysis highlights that these variables with significant correlations can predict each other’s spatial attributes. The annotated *p*-values and correlation coefficients provide a clear indication of the predictive power of SC and AC. The third subset evaluated the cognitive variables SE and SEE. The correlation analysis revealed the extent to which these cognitive measures were related. Significant correlations indicate a strong predictive relationship in which one cognitive measure can provide insights into another. The annotated statistical values emphasize the confidence levels of these relationships.

Each subset included the Pearson’s correlation coefficient (r) and *p*-values as annotations; statistical significance is denoted as *p* < 0.05, *p* < 0.01, and *p* < 0.001. Significant correlations highlight potential predictive relationships, whereas non-significant correlations (with no asterisks) suggest insufficient evidence for a linear predictive relationship between pairs of variables. The temporal analysis subsets (SRT and AT) indicated that these variables could significantly influence each other’s temporal dynamics. The spatial analysis subsets (SC and AC) revealed that spatial characteristics were interrelated and had potential predictive capabilities. The cognitive analysis subsets (SE and SEE) showed that cognitive measures were strongly correlated, suggesting that they could predict cognitive performance. Overall, the presence of significant correlations in these analyses underscores the importance of considering these relationships in predictive modeling and further research (see Appendix A.2.3 for more details).

Next, we estimated the observed sample data for statistical testing using both traditional (descriptive statistics such as M and SD) and regression-based (regression coefficients) approaches to estimate the effect of physical activity participation on aging and to provide a self-diagnostic profile based on these estimates. Despite individual differences, we performed statistical estimation and prediction using the measured variables in both male and female subjects without cognitive issues in order to establish the age-related effects of the Stroop task.

Table 4 presents the statistics for simple reaction time (SRT), anticipation time (AT), Symmetrical Spatial Coordination Ability (SC), Asymmetrical Spatial Coordination Ability (AC), and SE. The analysis of M and SD for each variable showed that the average SRT was 7.93 s, and the AT was 2.51 s. The average index for SC was 1.34 on average, and that for AC was 0.36 on average. The average time for SE, representing executive function, was 82.31 s, and the average frequency for SEE was 9.08. The SDs for each variable were 0.84 s for SRT, 1.10 s for AT, 0.21 for SC, 0.24 for AC, 35.67 s for SE, and 6.31 for SEE. These results suggest diversity between time- and space-based coordination abilities and executive function during the aging process. Specifically, the variability in SE and SEE was interpreted as reflecting individual differences in cognitive function decline. (See Appendix A.3 for more details.)

**Figure 5:**
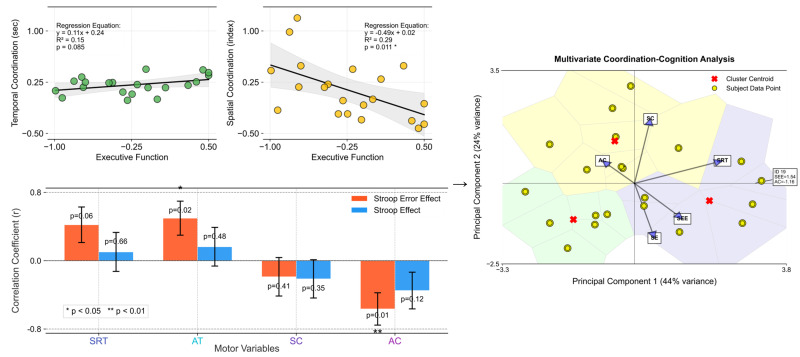
Outcomes for time-based coordination (measured in seconds) and space-based coordination (indexed values) as a function of executive function (averaged SE and SEE scores). The upper left set: The left plot displays temporal coordination against executive function, showcasing a linear relationship with a regression line (black). The right plot delineates spatial coordination vs. executive function, also highlighted with a corresponding regression line. Both plots include scatter points (yellow with black edges) representing individual data points, set against a grid for enhanced readability. The bottom left set: The plot depicts the correlation coefficients between motor variables (SRT, AT, SC, AC) and executive functions (SEE, SE). The bars represent the correlation coefficients. Red bars correspond to correlations with SEE, while blue bars represent correlations with SE. Asterisks (*) denote statistical significance (*p* < 0.05, with an error bar). The right set: This presents the results of the multivariate coordination–cognition analysis using principal component analysis (PCA). This visualization maps participants (yellow circles) and variable contributions (blue vectors) in the reduced dimensionality space, with the first two principal components explaining 44% and 24% of variance, respectively. Color-coded regions represent clusters of similar coordination–cognition patterns, with red X markers indicating cluster centroids. The vectors’ directions and lengths illustrate the relative contribution of each motor (SRT, AC, SC) and cognitive (SEE, SE) variable to the principal components, highlighting the strong relationship between asymmetric coordination and executive function measures.

In Figure 5, the regression analysis shows a more pronounced correlation between executive function and space-based coordination than time-based coordination. This is evident from the slopes and positions of the regression lines in both coordinations. A steeper slope in the spatial coordination signifies a stronger relationship, indicating that as executive function improves, the impact on spatial coordination abilities is more substantial. This finding suggests that interventions aimed at enhancing executive functioning may yield more substantial improvements in spatial coordination tasks than in temporal ones. This analysis provides insightful evidence of the differential impact of executive function on temporal versus spatial motor coordination.

The stronger correlation observed for spatial coordination suggests a more integral role for executive cognitive processes in spatial task performance, highlighting the complexity and cognitive demands of spatial motor skills. This relationship emphasizes the cognitive–motor interface, where cognitive improvements can lead to enhanced motor coordination, particularly in tasks requiring spatial awareness and processing. This connection may reflect the cognitive demands of motor planning and execution, where higher executive dysfunction affects motor performance more profoundly. This, in turn, suggests a potential avenue for targeted interventions aimed at enhancing motor coordination through cognitive training, particularly focusing on reducing complex errors (SEE), which are strongly correlated with motor performance.

**Figure 6:**
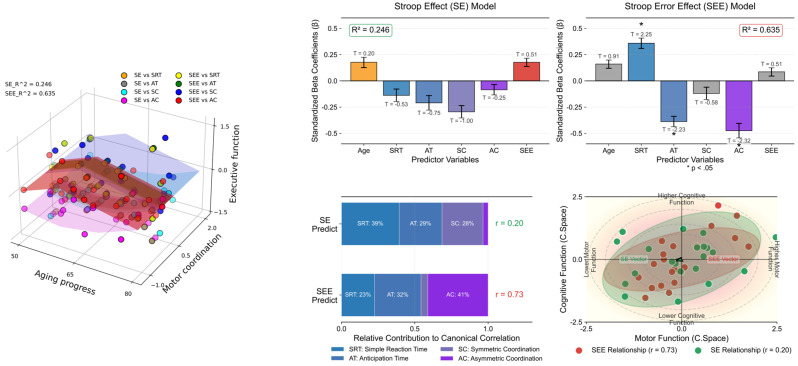
Comparative analysis with multiple-regression models. The left panel 3D scatter plot illustrates the correlation between age, motor coordination (SRT, AT, SC, AC), and executive functions (SE and SEE), color-coded by task type. The upper right panel illustrates the coefficients and their standard errors (vertical error bar) for predicting Stroop effects (**left**), highlighting no significant predictors (R^2^ = 0.246), and Stroop error effects (**right**), with asterisks indicating variables (*p* < 0.05) signifying a statistically significant predictive relationship (R^2^ = 0.635). T-values are annotated above the corresponding bars to provide insights into the direction and magnitude of each variable’s effect. The left panel on the bottom right displays the relative contributions of motor variables (SRT, AT, SC, AC) to predicting executive functions (SEE and SE), with canonical correlation coefficients shown. The right panel visualizes the canonical space relationship between motor function (x-axis) and cognitive function (y-axis), featuring data points with confidence ellipses and directional vectors that demonstrate the strength and direction of these relationships. The visualization effectively illustrates how motor coordination variables collectively relate to executive performance measures in canonical space.

Table 5 and Figure 6 present the results of the multiple-regression analyses exploring the impact of spatial coordination and other variables on Stroop test performance, measured using interference time and error effects (See Appendix A.4 for more details). We conducted separate analyses for the two dependent variables: SE and SEE. For SE (R^2^ = 0.246), none of the predictor variables, including age, SRT, AT, SC, AC, and SEE, showed significant predictive value for Stroop interference time, as indicated by their *p*-values being above the significance threshold (*p* < 0.05). Conversely, the SEE model, which explained a larger proportion of the variance (R^2^ = 0.635), identified several significant predictors. Specifically, SRT positively predicted SEE (β = 0.359, *p* = 0.038), which indicates that longer simple reaction times are associated with more errors. AT negatively predicted the SEE (β = −0.388, *p* = 0.040), which suggests that quicker reaction times are linked to fewer Stroop test errors. Similarly, AC showed a significant negative relationship with SEE (β = −0.475, *p* = 0.033), which implies that better coordination correlates with reduced errors. Age, SC, and SE did not significantly predict SEE.

These results highlight the complex interplay between various cognitive and perceptual–motor skills that influence Stroop test performance, particularly error propensity. Specifically, the reaction time and coordination abilities (both auditory and attentional) significantly affected the likelihood of errors in the Stroop task, underscoring the multifaceted nature of cognitive interference and executive function.

## 4. Discussion

This study revealed a significant negative association between asymmetric spatial coordination (AC) and Stroop error effects (SEEs) (r = −0.475, *p* = 0.033) in community-dwelling older adults, with AC emerging as the strongest predictor in the regression model (β = −0.475, R^2^ = 0.635). These findings align with and extend previous work on motor–cognitive relationships. The stronger association of spatial coordination measures (particularly AC) with cognitive errors, compared to temporal measures, suggests that the ability to independently control bilateral limb movements may share underlying processes with executive inhibition. The asymmetric coordination task requires suppression of the natural tendency toward symmetric movement—a demand conceptually similar to the inhibition required in incongruent Stroop trials.

This study empirically supports the hypothesis that cardiovascular and coordination exercises are closely related to cognitive function. This context aligns with studies on adults with mild brain damage that focus on visuomotor processes and working memory tests (Mulder et al., 2002), and behavioral cross-sectional studies on preschool children that have found positive relationships between coordination exercise and cognitive abilities (Planinsec, 2002). The observation that coordination exercise ability is highly correlated with executive-function-related areas (parietal and inferior frontal cortices) in older adults at a neurophysiological level (Voelcker-Rehage et al., 2010) is significant. This suggests that coordination exercise tasks (e.g., lines and obstacles) can help with the sprouting and restructuring of neural synapses in the aging brain (Black et al., 1990; Smiley-Oyen et al., 2008), which provides empirical validation for the proposition and highlights the observed significant relationships between asymmetrical-coordination-exercise functions and the older adult population.

**Figure 7:**
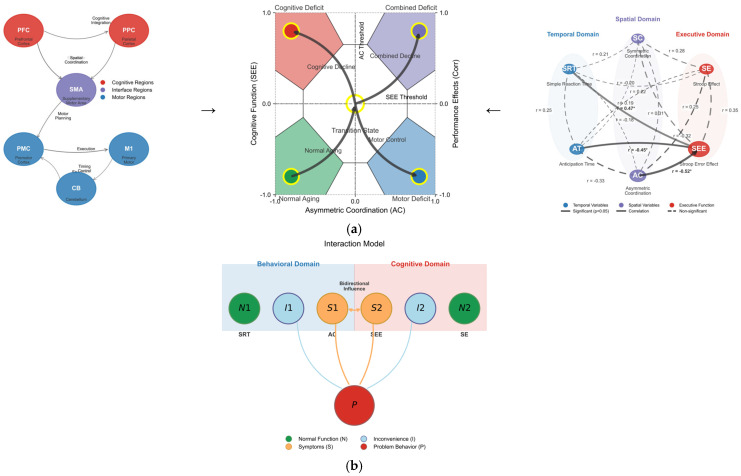
(**a**) Comprehensive model of motor–cognitive interactions in aging. Left set: Neural pathway diagram showing brain regions supporting motor–cognitive control. Middle set: Behavioral-cognitive interaction model where S denotes the conflicting symptom (AC in motor domain, SEE in cognitive domain); P represents a problem behavior of older adults (symptoms of the problem); I is the inconvenience derived from P; and N denotes normal behavior (SRT, SE, which must be non-noticeable for the tests but considered a typical symptom). Right set: Correlation network visualizing the relationships between temporal, spatial, and executive variables with varying strengths. (**b**) Comprehensive illustration of interconnected interaction patterns. Symptom space mapping demonstrating how asymmetric coordination (AC) and the Stroop error effect (SEE) define four distinct functional states with transition pathways between them. The integrated model demonstrates how motor coordination abilities interact with and predict cognitive performance in aging, with asymmetric coordination serving as a critical predictor of executive function. Asterisks (*) denote statistical significance.

First, the physical location capabilities of the older adult brain are related to the prefrontal cortex and parietal/posterior cingulate cortex areas (Maguire et al., 1999) and are associated with the frontoparietal network (S. J. Colcombe et al., 2004, 2006; Marks et al., 2007), which suggests a close relationship with physical and cognitive activities in daily life (Hillman et al., 2008). The reasons for the changes in cognitive function due to physical activity should be further examined from a neurophysiological perspective. Exercise (i.e., cardiovascular training) facilitates angiogenesis (Black et al., 1990) and results in plasticity in new neurons or neurotransmitter systems (van Praag et al., 2005). Specifically, coordination tasks assist in the formation or reorganization of synapses (Black et al., 1990). The close relationship observed between asymmetrical coordination movements and cognitive function indicators in this sample suggests that better coordination performance may reflect preserved neural function (Cotman et al., 2007; Vaynman & Gomez-Pinilla, 2006). Finally, while exercise does not generally stimulate cognitive function, it does affect specific stages of information processing, such as the sensorimotor process (Audiffren, 2008). Coordination tasks may engage similar neural processes to cognitive tasks, which aligns with the hypothesis that exercise influences neurotransmitter activity, potentially enhancing task performance (Ferrer-Uris et al., 2022; McMorris, 2008). The reaction times and coordination exercise tasks measured in this experiment were related to the premotor cortex and supplementary motor areas, suggesting a model (profile) that could empirically complement the interrelations in these prior studies (Cotman et al., 2007; Vaynman & Gomez-Pinilla, 2006) as follows:

Figure 7a,b depicts the complex interactions between problematic behaviors observed in the older adult population, discomfort arising from these behaviors, and normal behaviors. S represents conflicting symptoms corresponding to the SEE and AC, which are of primary interest. P indicates problematic behaviors exhibited by older adults as symptoms of the issue, while I signifies the various discomforts stemming from these problematic behaviors. N refers to normal behavior, reflecting typical symptom types such as SRT and SEs. Understanding these interrelations through motor and neurological analyses may aid in creating a self-diagnostic profile of aging. First, a mismatch in asymmetric bi-manual coordination (symptom) is associated with SEE, which may indicate a decline in cognitive function. Second, discomfort (behavioral and cognitive) originates from problematic behaviors that degrade the quality of daily life, thus necessitating improvement. Third, normal behavior appears when spatial and temporal actions, along with cognitive function, are within the normal range, which should be considered a typical symptom. Finally, the mismatch between asymmetric bi-manual coordination and SEE can serve as an indicator of problematic behavior, providing essential information for cognitive and behavioral interventions in older adults (Table 6 and Table 7).

The self-diagnostic profile presented above was designed to help individuals easily assess their cognitive and motor coordination status and take the necessary actions. It is structured around five indicators: asymmetric bi-manual coordination ability, SEE, discomfort in daily activities, normal behavior, and problem behavior (See Appendix A.5 for more details of their interrelations). Each indicator describes the respective state, indicates potential concerns, and recommends appropriate actions. For example, difficulties in asymmetric bi-manual coordination can be seen as a sign of cognitive function decline, and consultation with a professional is recommended. The SEE serves as an indicator of cognitive interference, suggesting the need for cognitive and motor evaluations. Discomfort in daily activities can affect one’s quality of life, necessitating strategies for improvement through therapy or lifestyle changes, and actions are proposed for both normal and problem behaviors.

Physical activity in older adults positively affects various aspects of life, including their quality of life (Field et al., 2001; Hillman et al., 2008; Kim et al., 2007; Lindner, 2002). It positively affects all areas, including perceptual skills, intelligence quotient, achievement, verbal and math tests, and developmental levels (Sibley & Etnier, 2003). Specifically, complex coordination movements correlate highly with executive function (Castelli et al., 2007), which suggests that physical activity in old age is crucial for developing or maintaining cognitive functions (Hillman et al., 2008). This study aimed to elucidate the relationship between cognitive function and physical activity based on previous cognitive-motor research in older adults (Koulouri et al., 2022) in order to provide an effective self-diagnostic method for measuring cognitive function and offer a direct profile prototype for this population.

Although our findings indicate a positive relationship, particularly with executive functions, it is important to acknowledge several limitations. First, the sample size (n = 24) relative to the number of predictors in the regression models warrants caution in interpretation. While the participant-to-predictor ratio falls below commonly recommended thresholds (e.g., 10:1), the significant findings for AC, SRT, and AT predicting SEE provide preliminary evidence for targeted investigation in larger samples. The R^2^ value of 0.635 should be interpreted as potentially optimistic; future studies with larger samples should confirm these relationships. Second, the cross-sectional nature of the study makes it difficult to infer causality; therefore, future research should consider intervention studies and longitudinal designs to track cognitive changes over time. Third, this study did not control for potential confounders, including cognitive reserve (e.g., occupational complexity, lifetime intellectual engagement), color vision deficits, visual acuity, or depression, which may influence both motor coordination and Stroop test performance. Fourth, while formal sensitivity analyses were not conducted, the exclusion rate (12.5%) is within an acceptable range for reaction time studies (Ratcliff, 1993), and detailed outlier criteria are reported in Appendix A. In addition, integrating more quantitative measures of cognitive and motor abilities could refine our understanding of how specific types of physical activity contribute to cognitive resilience in an aging population. This would pave the way for tailored exercise programs that support cognitive health and quality of life among older adults.

## 5. Conclusions

Advancements in foundational research and technology necessary to understand and address issues related to aging have occurred across various fields such as medicine and cognitive science (brain imaging techniques, fMRI, and qualitative research methods). At the same time, artificial intelligence technologies needed to implement aging-related services through internet technology (IT) (Turnbull et al., 2022) have been developed. Integrated products that combine medical, behavioral psychology, education, and rehabilitation science have emerged (e.g., neurofeedback and therapy using virtual reality), particularly in the IT-based older adult cognitive rehabilitation service industry (e.g., SoftTools and Fast Forward). The industry has been formally developing since the 20th century, with a growing number of products and services centered on “wellness” and “anti-aging” concepts (Brink, 2023). Federal and state governments provide age-related information through various companies that participate in the formation of senior-related business models. Products mindful of older age groups are being launched, including functional games for older adults that activate dormant senses or abilities through direct actions to provide enjoyment and train the various capabilities required in daily life (Lau & Agius, 2021).

We also recognize the progressive aging situation. As the cognitive-related industry begins to receive attention, related products are being released, although they are still in the initial formation stage (e.g., the “Youthful Village” for dementia-related cognitive improvement, and the “Experiential Bicycle Game” being developed by several universities to enhance the physical strength and mobility of older adults). In particular, regarding cognitive measurement technology, programs remain limited to commercial levels for aptitude screening (e.g., memory and concentration tests for commercial drivers used by the Traffic Safety Corporation) and do not precisely match actual realities (Hoffman & Robertson, 2024). The content is not fully verified and remains at a simple level, offering only entertainment and route memorization. This reflects the rapid market growth of older-adult-related industry products and services owing to this population’s continuous increase through the Elderly-Friendly Industry Promotion Project (Gargon et al., 2014). However, sufficient discussion is not taking place. Specific diagnostic guidelines to address the complex interactions of various factors (psychological state, nutritional status, and lifestyle habits) targeting older adults (Barbour & Blumenthal, 2005) have not been established (Hillman et al., 2008).

Therefore, this study aimed to comprehensively characterize motor control abilities in older adults based on motor and cognitive control traits found in existing experiments and research. The findings can be employed to establish a detailed approach to assessing the neuromotor control traits that develop during aging by considering how older adults’ integrated motor control abilities affect the use of cognitive functions (Nshimiyimana et al., 2023). Second, we sought to develop a self-diagnostic integrated measurement technology to evaluate neuromotor control traits in older adults. Traditionally, developing a detailed understanding of the characteristics of neurological and motor control in older adults requires equipment, time, and human resources (Greenhalgh et al., 2013), which presents difficulties in terms of daily access, complexity, and high costs. Developing a unified measurement technology that allows for the self-diagnosis of aging-related neuromotor information using complex and diverse measurement equipment technologies will make it easier to assess and address aging-related diseases.

In summary, this cross-sectional study provides preliminary evidence for associations between motor coordination abilities and cognitive function in older adults. Asymmetric spatial coordination showed the strongest association with Stroop error effects, suggesting potential utility as a component of self-assessment tools. The proposed study should be considered a proof-of-concept requiring validation in larger, longitudinal studies before clinical application.

## Figures and Tables

**Table 1:** Categorization of the experimental motor coordination tasks.

Motor Tasks	Category
**Simple reaction time/anticipation time** **Symmetric/asymmetric drawing**	Time-based speed and accuracy Space-based coordination

Note. Referenced from Beek and Turvey (1992); Pelz et al. (2001); Rose and Winstein (2013).

**Table 2:** Descriptive statistics for each variable.

Id	Sex	Age	Time-Based Coordination		Space-Based Coordination	
			SRT (s)	AT (s)	SC (Index)	AC (Index)
1	Female	71	7.36	4.20	1.10	0.05
:	:	:	:	:	:	:
24	Female	80	6.20	1.32	1.54	0.51
Averaged (M)			7.93	2.51	1.34	0.36
Variation (SD)			0.84	1.10	0.21	0.24

Note. SRT = simple reaction time, AT = anticipation time, SC = symmetric spatial coordination, and AC = asymmetric spatial coordination. According to the cutoff score, three participants were considered outliers based on quartile deviation above the mean (Ratcliff, 1993), and they were eliminated. Exemplar participant data are shown; complete individual data available in Appendix A.2.1 to support reproducibility.

**Table 3:** Descriptive statistics for each variable.

Id	Sex	Age	Executive Function	
			SE (s)	SEE (Freq)
1	Female	71	88.16	7.5
:	:	:	:	:
24	Female	80	130.7	6.5
Averaged (M)			82.31	9.08
Variation (SD)			35.67	6.31

Note. SEE = Stroop error effect, and SE = Stroop effect. See Appendix A.2.2 for details on participants’ scores.

**Table 4:** Descriptive statistics for each variable.

Id	Sex	Age	Time-Based Coordination		Space-Based Coordination		Executive Function	
			SRT (s)	AT (s)	SC (Index)	AC (Index)	SE (s)	SEE (Freq)
1	Female	71	7.36	4.20	1.10	0.05	88.16	7.5
:	:	:	:	:	:	:	:	:
24	Female	80	6.20	1.32	1.54	0.51	130.7	6.5
Averaged (M)			7.93	2.51	1.34	0.36	82.31	9.08
Variation (SD)			0.84	1.10	0.21	0.24	35.67	6.31

Note. SRT = simple reaction time, AT = anticipation time, SC = symmetric spatial coordination, AC = asymmetric spatial coordination, SEE = Stroop error effect, and SE = Stroop effect. See Appendix A.3 for all details on participants’ scores.

**Table 5:** Multiple-regression analysis results for spatial coordination predicting the Stroop test interference time.

Variable	β	T	Significant t	R^2^
Dependent Variable: Stroop Error Effects				0.635
SRT	0.359	2.250	0.038 *	
AT	−0.388	−2.230	0.040 *	
AC	−0.475	−2.321	0.033 *	

Note. * *p* < 0.05; SRT = simple reaction time, AT = anticipation time, and AC = asymmetric spatial coordination. Using the regression-based approach, the raw scores of each independent variable were normalized to Z scores. The dependent variable was the Stroop interference score. (See Appendix A.4 for details on the multiple-regression results.)

**Table 6:** Self-check profile for cognitive and motor coordination.

Indicator	Description	Potential Concern	Action Suggestion
Asynchronous bi-manual coordination	Difficulty performing tasks that require both hands to work together in a coordinated manner	Sign of cognitive decline	Consult a healthcare professional for a detailed assessment
Stroop error effect	Errors or increased time needed to distinguish colors or words on a Stroop test	Indicator of cognitive interference	Engage in cognitive exercises and consider cognitive assessments
Behavioral inconvenience	Challenges in daily activities due to changes in motor coordination or cognitive functions	Quality of life may be affected	Seek strategies for improvement through therapy or lifestyle changes
Normal behavior	Consistent performance in tasks requiring spatial (temporal) behavior and cognitive function without noticeable difficulty	No immediate concern but continue monitoring	Maintain a healthy lifestyle and regular cognitive activities
Problem behavior	Notable difficulty in tasks requiring asymmetrical bi-manual coordination or cognitive tasks, as indicated by Stroop test errors	Sign of cognitive or motor function decline	Professional consultation and possibly cognitive or physical therapy

Note. Participants were given the following instructions: Check if you experience any of the indicators described in the table. Determine the potential concern associated with these indicators. Follow the suggested actions, whether by consulting a healthcare professional, engaging in specific exercises, or monitoring changes over time.

**Table 7:** Cognitive and motor coordination self-check sheet.

Indicator	Statement	Checklist		
		High	Middle	Low
Asynchronous bi-manual coordination	I find it difficult to perform tasks that require both hands to work together.	[ ]	[ ]	[ ]
	My ability to coordinate movements between my hands has decreased.	[ ]	[ ]	[ ]
Stroop error effect	I experience confusion or delays when naming the color of words.	[ ]	[ ]	[ ]
	I make frequent errors or take longer to complete color-word distinguishing tasks.	[ ]	[ ]	[ ]
Behavioral inconvenience	Challenges in daily activities are noticeable due to coordination or cognitive changes.	[ ]	[ ]	[ ]
	Activities that used to be easy now require more effort.	[ ]	[ ]	[ ]
Normal behavior	I perform tasks requiring spatial-temporal behavior and cognitive function without difficulty.	[ ]	[ ]	[ ]
	My daily activities are not hindered by changes in coordination or cognitive abilities.	[ ]	[ ]	[ ]
Problem behavior	I have difficulty with tasks requiring asymmetrical bi-manual coordination.	[ ]	[ ]	[ ]
	Signs of cognitive or motor function decline affect my daily life.	[ ]	[ ]	[ ]

Note. Participants were given the following instructions: High indicates frequent experiences of the stated conditions, which suggests a need for further professional evaluation. Middle may warrant monitoring and possibly preventative measures. Low means rare or no experiences of the stated conditions, in which case the individual should maintain a healthy lifestyle and regular monitoring. Note that this is a preliminary tool for self-awareness. If you have concerns—especially if you have checked several items as high—consulting a healthcare professional is strongly recommended.

**Figure A1:**
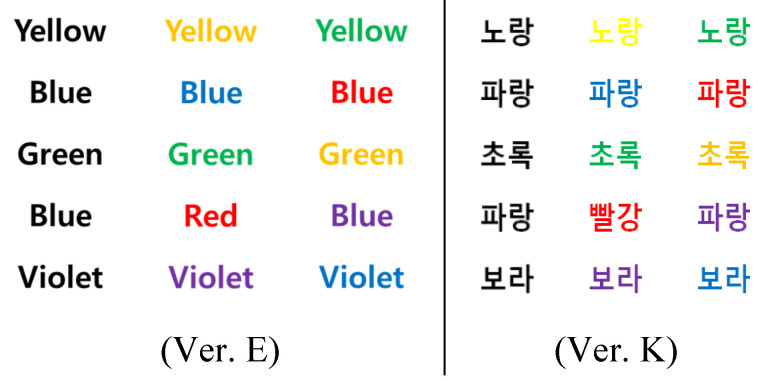
Each set of prototypes (left set = English version, right set = Korean version) in the Stroop task: left = Stroop 1 (reading the word), middle = Stroop 2 (reading the color), right = Stroop 3 (reading the color, not the word) (referenced from Keele, 1972; Hecht & Reiner, 2010).

**Figure A2:**
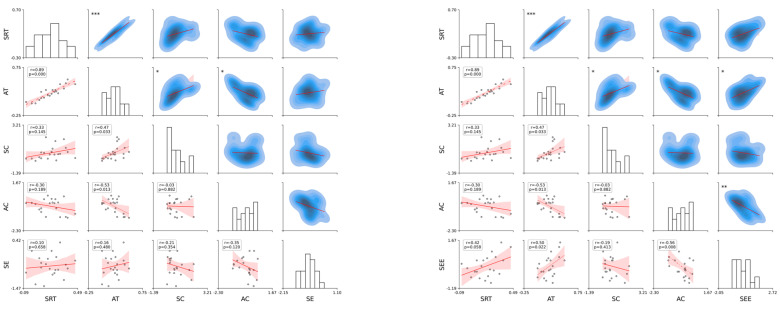
Each subplot shows the linear regression line with Pearson’s correlation coefficients (r) and *p* values, indicating the strength and statistical significance of the relationships. The scatter plots illustrate the pairwise relationship, and asterisks on the kernel density estimation (KDE) plots in the upper triangle denote the significance levels *p* < 0.05, *p* < 0.01, and *p* < 0.001. The style of the regression lines varies according to the significance, assisting in visualizing the correlation strength between variable pairs. The presence of significant correlations (denoted with asterisks) highlights potential predictive relationships, whereas non-significant correlations (no asterisks) indicate a lack of evidence for a linear predictive relationship between the respective pairs of variables.

**Figure A3:**
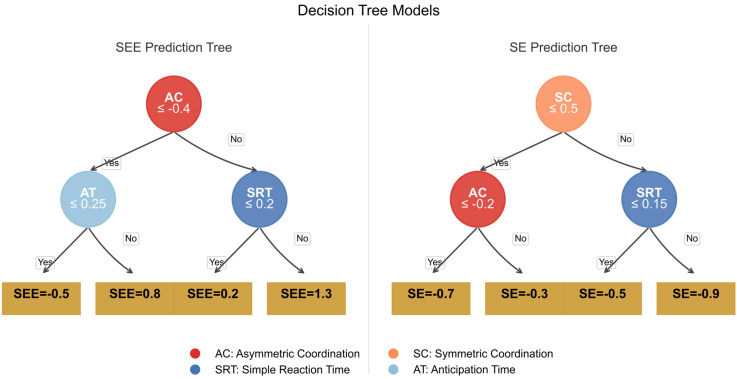
The image presents two decision trees side-by-side for predicting the target variables SEE (**left panel**) and SE (**right panel**), both using the same set of predictors: SRT, AT, SC, and AC.

## Data Availability

The data presented in this study are available on request from the corresponding author.

## Appendix A

### Appendix A.1. Asymmetry Bi-Manual Coordination Modeling

The potential of the difference between the asymmetry or spatially uncoupled bi-manual rhythmic components can be modeled as

$$\Delta \omega =\left(\omega_{L}-\omega_{R}\right)$$

where ω is the movement of the left ($\omega_{L}$) and right ($\omega_{R}$) individuals. If the relative phase between $\omega_{L}$ and $\omega_{R}$ were identical (Δω = 0), this pattern would be assumed to exhibit perfect symmetry. However, as the preferred movements of the individual oscillators are not the same (i.e., detuning), the expected stability of the rhythmical limb oscillation dynamics is no longer equal. Such a symmetry-breaking phenomenon is a fundamental feature of the coordinative system. From this dynamic, the different noise levels of the underlying subsystems (neural, muscular, and vascular) can be estimated around an equilibrium point. When conceptualizing the model, the operational definitions of each category are made with respect to which model has to consider the variability of the relative phase frequencies between two limbs:

$$\dot{\phi }=\Delta \omega -\alpha \;cos\left(\phi \right)-b\;cos\left(2\phi \right)+\sqrt{\varrho \xi_{t}}$$

The estimation between two oscillators’ relative traces ($\dot{\phi }$) is captured by the parameter (Δω) of the preferred movement of the individual segment [$\alpha \;cos\left(\phi \right)-b\;cos\left(2\phi \right)$] with the noise ($\sqrt{\varrho \xi_{t}}$). Given the equation of the preceding model (grouped as the kinematics of motor stability according to the coordination task of synchronization), this term has been used to capture purely functional dynamics regarding the equilibria and is usually confirmed by the time and temporal differences between an oscillating limb (α and b).

### Appendix A.2. Experimental Cognitive Task

#### Appendix A.2.1

**Table A1:** A summary of descriptive statistics pertaining to the motor coordination variable. The measures used to tap the two-target executive function (inhibition) indicators are presented in the table below.

Id	Sex	Age	Time-Based Coordination		Space-Based Coordination	
			SRT (s)	AT (s)	SC (Index)	AC (Index)
1	Female	71	7.36	4.20	1.10	0.05
2	Female	72	8.24	1.69	1.39	0.47
3	Female	72	9.23	1.81	1.20	0.33
4	Female	73	8.51	1.83	1.30	0.24
5	Male	73	7.57	1.37	1.15	0.04
6	Female	78	6.67	2.45	1.58	0.5
7	Female	78	7.96	1.38	1.30	0.33
8	Female	79	7.98	2.98	1.25	0.21
9	Female	80	8.75	1.46	1.14	0.15
10	Female	80	6.72	1.29	1.60	0.5
11	Male	81	8.38	2.51	1.55	0.52
12	Male	81	8.21	2.60	1.10	0.04
13	Male	81	6.30	1.67	1.05	0.29
14	Female	82	8.14	3.14	1.38	0.02
15	Female	84	7.94	4.05	1.47	0.52
16	Female	84	7.49	1.30	1.63	0.75
17	Female	85	8.08	2.42	1.60	0.5
18	Female	87	8.16	4.13	1.70	0.63
19	Female	87	8.87	4.64	1.50	0.72
20	Female	89	8.95	4.28	1.23	0.05
21	Female	83	7.71	2.88	1.20	0.63
22	Male	78	9.16	1.70	1.07	0.05
23	Male	82	7.84	3.18	1.15	0.64
24	Female	80	6.20	1.32	1.54	0.51
Averaged (M)			7.93	2.51	1.34	0.36
Variation (SD)			0.84	1.10	0.21	0.24

Note. According to the cutoff score, three participants were considered outliers based on quartile deviation above the mean, and they were eliminated. See Appendix A.1 for details on participants’ scores. SRT = simple reaction time, AT = anticipation time, SC = symmetric spatial coordination, and AC = asymmetric spatial coordination.

#### Appendix A.2.2

**Table A2:** A summary of descriptive statistics pertaining to the cognitive function variables. The measures used to tap the two-target executive function (inhibition) indicators are presented in the table below.

Id	Sex	Age	Executive Function	
			SE (s)	SEE (Freq)
1	Female	71	88.16	7.5
2	Female	72	23.13	2.5
3	Female	72	110.86	14
4	Female	73	58.15	20.5
5	Male	73	54.26	21.5
6	Female	78	72.84	1
7	Female	78	92.16	5
8	Female	79	77.49	10
9	Female	80	99.47	16
10	Female	80	60.66	8.5
11	Male	81	69.56	5.5
12	Male	81	148.05	11.5
13	Male	81	107.13	5
14	Female	82	74.19	10
15	Female	84	54.69	3
16	Female	84	105.2	10
17	Female	85	59	6
18	Female	87	68.96	0
19	Female	87	36.98	4
20	Female	89	86.49	18
21	Female	83	17.63	2
22	Male	78	149.04	19
23	Male	82	130.64	11
24	Female	80	130.7	6.5
Averaged (M)			82.31	9.08
Variation (SD)			35.67	6.31

Note. SEE = Stroop error effect, and SE = Stroop effect. According to the cutoff score, three participants were considered outliers based on quartile deviation above the mean, and they were eliminated. One participant scored 366.04 s (*Z*: 4.10) on the SE, another scored 33.5 f (*Z*: 2.53) on the SEE, and the last scored 36.5 f (*Z*: 2.85) on the SEE. As our data can be distorted by a greatly increased range, we considered that these three participants were not from the same population.

### Appendix A.3

**Table A3:** A summary of descriptive statistics pertaining to all the variables. The measures used to tap the two-target executive function (inhibition) indicators are presented in the table below.

Id	Sex	Age	Temporal-Based Coordination				Spatial-Based Coordination		Executive Function	
			SRT (s)	AT (s)			SC (Index)	AC (Index)	SE (s)	SEE (Frequency)
1	Female	71	7.36	4.20			1.10	0.05	88.16	7.5
2	Female	72	8.24	1.69			1.39	0.47	23.13	2.5
3	Female	72	9.23	1.81			1.20	0.33	110.86	14
4	Female	73	8.51	1.83			1.30	0.24	58.15	20.5
5	Male	73	7.57	1.37			1.15	0.04	54.26	21.5
6	Female	78	6.67	2.45			1.58	0.5	72.84	1
7	Female	78	7.96	1.38			1.30	0.33	92.16	5
8	Female	79	7.98	2.98			1.25	0.21	77.49	10
9	Female	80	8.75	1.46			1.14	0.15	99.47	16
10	Female	80	6.72	1.29			1.60	0.5	60.66	8.5
11	Male	81	8.38	2.51			1.55	0.52	69.56	5.5
12	Male	81	8.21	2.60			1.10	0.04	148.05	11.5
13	Male	81	6.30	1.67			1.05	0.29	107.13	5
14	Female	82	8.14	3.14			1.38	0.02	74.19	10
15	Female	84	7.94	4.05			1.47	0.52	54.69	3
16	Female	84	7.49	1.30			1.63	0.75	105.2	10
17	Female	85	8.08	2.42			1.60	0.5	59	6
18	Female	87	8.16	4.13			1.70	0.63	68.96	0
19	Female	87	8.87	4.64			1.50	0.72	36.98	4
20	Female	89	8.95	4.28			1.23	0.05	86.49	18
21	Female	83	7.71	2.88			1.20	0.63	17.63	2
22	Male	78	9.16	1.70			1.07	0.05	149.04	19
23	Male	82	7.84	3.18			1.15	0.64	130.64	11
24	Female	80	6.20	1.32			1.54	0.51	130.7	6.5
Mean		7.93			2.51		1.34	0.36	82.31	9.08
Standard deviation		0.84			1.10		0.21	0.24	35.67	6.31

Note. SRT = simple reaction time, AT = anticipation time, SC = symmetric spatial coordination, AC = asymmetric spatial coordination, SEE = Stroop error effect, and SE = Stroop effect. Initially, 30 participants volunteered to participate in this experiment (female = 20, male = 10), but we eliminated six participants [3 opted out (two males, one female), 3 were outliers (two males, one female)]. Thus, the total data we analyzed consisted of 24 sets. (See the explanation given in the manuscript.) Specifically, according to the exclusion criteria score, which identified outliers based on quartile deviation above the mean, we decided to delete three data points. One participant scored 366.04 s (*Z*: 4.10) on the SE, another scored 33.5 *f* (*Z*: 2.53) on the SEE, and the last scored 36.5 *f* (*Z*: 2.85) on the SEE. Because our data can be distorted by a greatly increased range, we considered that these three were not from the same population.

### Appendix A.4

**Table A4:** Multiple-regression analyses for spatial coordination predicting the Stroop Test interference time.

Variable	β	T	Significant t	*R* ^2^
Dependent variable: Stroop effects				**0.246**
Age	0.177	0.196	0.452	
SRT	−0.138	−0.532	0.602	
AT	−0.210	−0.751	0.463	
SC	−0.295	−1.002	0.330	
AC	−0.085	−0.253	0.803	
SEE	0.177	0.512	0.518	
Dependent variable: Stroop error effects				0.635
Age	0.160	0.909	0.376	
SRT	0.359	2.250	0.038 *	
AT	−0.388	−2.230	0.040 *	
SC	−0.120	−0.576	0.572	
AC	−0.475	−2.321	0.033 *	
SE	0.086	0.512	0.615	

Note. * *p* < 0.05; SRT = simple reaction time, AT = anticipation time, SC = symmetric spatial coordination, AC = asymmetric spatial coordination, SEE = Stroop error effect, and SE = Stroop effect. Using the regression-based approach, the raw scores of each independent variable were normalized to Z scores. The dependent variable was the Stroop interference score.

### Appendix A.5. Predicting the Target Variables Using the Stroop Error Test

The decision nodes are color-coded according to the importance weights of the variables in each tree, with more vivid colors indicating a higher impact on the model’s decision-making process. The left panel predicting the Stroop error effect (SEE) uses variable AC (Accuracy) as the main splitting factor, highlighted with a bold red color, indicating its significance in error prediction. Nodes corresponding to SEE are shown in blue, marking their influence in error fluctuation predictions. The right panel predicting the Stroop effect (SE) highlights the AC variable again as a key indicator, represented in orange to distinguish it from the SEE model. Nodes associated with the SEE are marked with a distinct magenta color, showing the variance explained by these nodes. The data-driven simulation emphasizes the distinct roles of AC in both models, and the weights of each node are reflected in the proportional node size and color intensity. This visualization enables a clearer understanding of how feature importance and variable influence differ.
